# Medical residency match applicants undervalue factors that predict stress and burnout

**DOI:** 10.1080/10872981.2022.2109243

**Published:** 2022-08-09

**Authors:** Kaitlyn A. Kaiser, Heather C. Lench, Linda J. Levine

**Affiliations:** a Department of Psychological and Brain Sciences, Texas A&M University, College Station, Texas, USA; b Department of Psychological Science, and fellow, Center for the Neurobiology of Learning and Memory, University of California Irvine, Irvine, California, USA

**Keywords:** Residency, stress, burnout, rank decisions

## Abstract

In the medical residency match process, applicants’ ranking decisions are influenced by multiple factors related to training, geography, and lifestyle expectations. Ranking decisions directly impact match results, with implications for emotional outcomes such as happiness and stress. The present study explored the decision factors considered most important by applicants when creating rank order lists (ROLs), and how match outcomes and program factors predicted happiness, enthusiasm, stress, and life satisfaction. Senior medical students (*n* = 182) at a large public university in California completed surveys at three timepoints, spanning from shortly before Match Day to several months into PGY-1. Study findings support that both program-related (e.g., training quality, program size) and non-program-related (e.g., geography, work life balance) factors are important to applicants when making ROL decisions. Applicants who matched with their top choice program initially experienced emotional benefits, but these emotional differences did not persist into PGY-1, where all matched applicants had similar levels of emotion and life satisfaction. The emotional cost and benefits of matching with programs of different ROL positions (e.g., matching with top-choice program or not) were most apparent shortly after matching but in the long-term, a stronger predictor of PGY-1 emotions was perceived person-program alignment. Person-program alignment (e.g., call schedule, patient caseload) also predicted burnout in the first few months of a residency program. These findings show that, when applicants are making ranking decisions, they undervalue factors that predict stress and burnout during residency.

Every March on Match Day, senior medical students find out where they will complete their residency training, the culmination of a months-long application process facilitated by the National Resident Matching Program (NRMP). Match outcomes are impacted by metrics of applicant qualifications (e.g., United States Medical Licensing Examination scores, number of research experiences and publications), as well as ranking behaviors (e.g., ranking one program over another, considering the competitiveness of a match).

The Match’s underlying algorithm works optimally when applicants create their rank order lists (ROLs) based on their personal preferences [1]. However, analyses of unmatched applicants show evidence of strategically ranking or omitting programs from a ROL based on the perceived likelihood of matching [2], sometimes resulting in students failing to match who otherwise would have. Applicants who follow the recommended preference-based ranking strategy are less likely to leave Match Day with an unsatisfactory outcome [3], but there may be individual differences in how program preferences are formed. Previous studies have established that applicants consider a number of ranking factors including geographic location, training quality, and interview impressions [4–6], demonstrating that program preference is an amalgamation of multiple evaluations.

Our investigation aims to provide further insight into the decision-making process students engage in as they attempt to identify their preferred residency programs, and the relative importance they give to ranking factors. Many studies on ROL decisions focus on a particular specialty area [6–8] or analyze the importance of one specific ranking factor (e.g., diversity [7], program websites [9]). To assess decision-making more generally, the present study surveyed applicants across a wide range of specialty areas on ROL considerations, including program-related, geographic, and lifestyle factors.

A second aim of this investigation was to identify factors that actually were important to emotional responses as students learned the outcome of Match Day, as well as several months after beginning their residency program. We assessed respondents at three timepoints: shortly before Match Day, shortly after Match Day, and in the fourth month of their residency program.

## Methods

Senior medical students at a large public university in California were contacted to participate in the study with approval from the IRB. Several weeks before Match Day, students were invited by a classmate in student leadership to participate in a study that involved three online surveys. Students who elected to participate received $25 for completing the first survey, and $50 each for the second and third surveys. Two cohorts of students (the graduating classes of 2016 and 2017) were recruited for the study, and data collection ran from March 2016 to October 2017. This study was part of a larger investigation about emotional forecasting [10,11], and only relevant methods and procedures are reported here.

The first survey was sent out 2 weeks before Match Day, after students had submitted their ROLs, and completed by a week before Match Day. In the survey, participants rated their level of general happiness, excitement, stress, and life satisfaction from 1 (*not at all*) to 9 (*most extreme possible*). The selected states – happiness, excitement, and life satisfaction – were chosen because of their relevance to well-being [12], and stress was included because it is a common experience for residents [13] and people undergoing life transitions such as beginning a new job or moving [14]. Single-item measures were selected for survey brevity and because these measures have been repeatedly validated for measurement of emotion, stress, and life satisfaction outcomes [15–17]. Other survey items concerned ROL decision factors. Participants listed the five most important factors they considered when making their rank list. Answers were provided in an open-response format categorized by research team members into the following categories: location, training, program culture, relationship, prestige, post-residency opportunity, finances, or other reasons. These categories were developed through thematic analysis of responses with discrepancies resolved by the lead investigator. Participants also rated the importance of a set of factors (from the NRMP’s Match PRISM App) often considered during ROL decisions [6,7,18] from 1 (*not important*) to 9 (*extremely important*).

The second survey link was delivered the day after Match Day, with a completion deadline of one week later. Applicants reported the outcome of the Match (whether they matched, the matched program, and the ROL position of the program). They also reported their current perception of the alignment between their priorities and those of the program on a scale from 1 (*does not meet my needs at all*) to 9 (*perfectly suited to my needs*). These priority items (shown in Table 2) were pulled from the list of factors considered by applicants during ROL decisions used in the first survey, with two items excluded that were not relevant to residents (‘likelihood of matching’ and ‘gut feelings during interview visit’). They also rated their current responses of happiness, enthusiasm, stress, and life satisfaction using the same scale as at Time 1.

The third, final survey was administered in mid-October, selected because it was several months into post-graduate year one (PGY-1). Applicants again rated their responses of happiness, enthusiasm, stress, and life satisfaction using the same scale as at Time 1. They also rated the person-program alignment using the same scale as at Time 2. Participants also reported the level of burnout they were currently experiencing from 1 (*I enjoy my work. I have no symptoms of burnout*) to 5 (*I feel completely burned out and often wonder if I can go on. I am at the point where I may need some changes or may need to seek some sort of help)*. Single-item measures of burnout have been validated against longer scales such as the Maslach Burnout Inventory and are recommended for increasing response rates among physicians specifically [19].

Analyses were conducted using SPSS statistical software, and an alpha of 0.05 was considered statistically significant. The factors that participants considered when making ROL decisions was analyzed from 1) responses to an open ended prompt about the top five factors considered and these were examined as the percentage of participants who gave each category of response, and 2) ratings of the importance of each factor on a continuous Likert-type scale, which were summarized as mean rated importance and standard deviations. The impact of match outcome (i.e., matching with top choice, second choice, third choice, fourth or lower) on responses was analyzed using an Analysis of Variance (ANOVA), which permitted the examination of whether responses on continuous Likert-type scales differed among the categories of the rank of match. Because ANOVAs are omnibus tests, independent contrasts were conducted to compare each category included in the ANOVA. Whether responses related to the perceived alignment between the person and the program was also examined, using linear regression analyses to examine the degree to which continuous Likert-type ratings of person-program alignment predicted responses to the match outcome that were also rated on continuous Likert-type scales.

## Results

The sample (*n* = 182 who completed Time 1 out of 203 eligible participants, 89.6% response rate; 179 responded to Time 2; 167 responded to Time 3) included 86 males (47.5%) and 95 females (52.5%); one participant did not report gender. The average age of participants was 28 years (range 25–36). Most participants (174) matched with a residency program through the NRMP, representing 72 different residency locations throughout the nation. Two matched into specialty areas that do not use the NRMP, two did not match into any program, and one participant was matched with a residency program through the Supplemental Offer and Acceptance Program after Match Day. Of matched participants (*n* = 174), 94 matched with the program they ranked first, 33 with the second ranked program, 16 with the third ranked program, and 31 with the program ranked fourth or lower.

### Ranking factors

We analyzed factors participants considered when making ROL decisions in two ways. First, we examined the free text answers listed by survey respondents as the top five factors they considered when ranking programs. These responses were categorized (described in methods), and inter-rater agreement was high (agreeing on 97.6% of classifications). The top five decision factors are described in Table 1. Location was listed as the most important factor by the highest number of participants, followed by training factors, and program culture (17.6%). Responses categorized as ‘other’ include considerations of training length, schedule, and patient population characteristics.

**Table 1.** Medical residency applicants were asked to report the five factors that were most important to them in making decisions about their Rank Order List. These open-ended responses were categorized into eight categories. The numbers represent the percentage of participants who listed each decision factor as being 1st, 2nd, 3rd, 4th, or 5th most important in their decision-making process.

	Location	Training	Prog. Culture	Relations	Prestige	Post-residency opportunity	Finances	Other
1st	46.7	17.6	17.6	6.0	8.8	2.7	-	0.5
2nd	19.2	22.5	26.4	3.8	14.8	6.6	-	6.6
3rd	14.9	20.4	24.3	6.6	11.0	8.3	1.7	12.7
4th	8.6	26.4	23.6	2.3	8.6	11.5	5.2	13.8
5th	7.3	21.8	26.7	5.5	10.3	8.5	9.1	10.9

*Participants were asked to list the ‘five factors that were most important to you in making decisions about your ROL.’*

**Table 2.** Participants rated the importance of each of 24 factors in their decisions for Rank Order Lists on a 9-point scale from *not important* (1) to *extremely important* (9). Factors were combined in some cases into superordinate categories, presented in bold below. The numbers represent the mean (M) rated importance of each factor, accompanied by the associated Standard Deviation (SD).

Decision Factor	*M*	*SD*
**Location**	8.3	1.3
**Prestige – Reputation of program**	7.1	1.7
**Post-residency opportunity** (average of items below)	7.0	1.7
Career paths of recent program graduates	7.2	1.5
Quality of preparation for fellowship	6.9	2.2
**Program culture** (average of items below)	6.9	1.1
Gut feelings about overall goodness of fit with the program	8.4	1.2
Gut feelings during interview visit	8.1	1.4
Morale of house staff and current residents	8.0	1.3
Rapport with program leadership	7.5	1.5
Work/life balance	6.9	1.9
Program’s flexibility to pursue electives and interests	6.3	2.0
Size of patient caseload	6.1	1.9
Cultural/racial/ethnic/gender diversity at institution	5.6	2.6
Call schedule	5.1	2.2
**Relationship** (average of items below)	6.8	2.2
Support network in the area	7.0	2.4
Needs of partner, spouse, or family	6.7	2.8
**Training** (average of items below)	6.7	1.1
Quality of clinical training	8.2	1.1
Appropriate balance between faculty supervision and resident responsibility for patient care	7.2	1.6
Diversity of patient problems or procedures	7.0	1.7
Quality of hospital facilities	6.7	1.8
Research opportunities	6.0	2.4
Size of program	5.1	2.2
**Finances** (average of items below)	4.8	2.3
Cost of living in the area	5.0	2.5
Salary, benefits	4.6	2.5
**Likelihood of matching**	4.5	2.8

The second source of information about ranking considerations came from ratings of the importance of specific factors when making ROL decisions at Time 1 (see Table 2). Of the 24 factors, participants rated gut feelings about goodness of fit as the most important, followed by geographic location, and quality of clinical training. The least important factors were likelihood of matching, salary and benefits, and cost of living in the area.

### Responses to match outcomes

**Figure 1.**
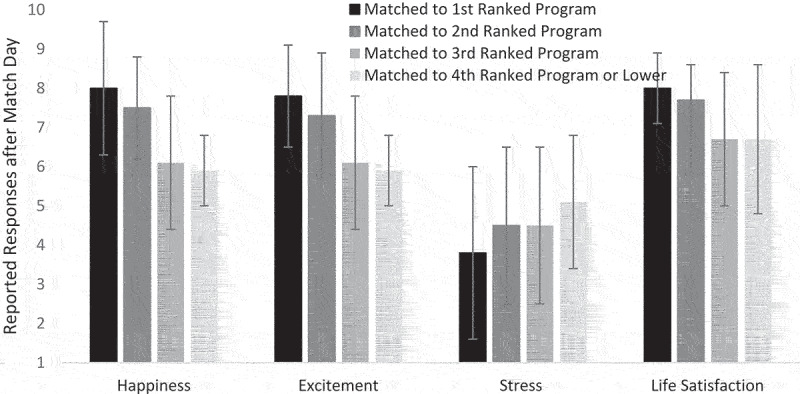
Participants rated the intensity of their responses of happiness, excitement, stress, and life satisfaction shortly after learning the outcome of Match Day on a 9-point scale from *not at all* (1) to *most extreme possible* (9). These responses are broken out in the figure according to whether they matched with their 1st choice, their 2^nd^ choice, their 3rd choice, or their 4^th^ or lower choice. Values represent the mean rated intensity of each response after Match Day, accompanied by the associated Standard Deviation.

The repeated measurement nature of the study permitted analyses of the relationship between ROL outcomes (of matching with a top choice or lower) and predicted responses such as happiness, enthusiasm, stress, and life satisfaction. Only two respondents did not match with any program, and this group was too small to analyze. To investigate the emotional impact of different match outcomes, Analyses of Variance (ANOVA) compared responses at Time 2 (shortly after Match Day) among applicants who matched into a program they ranked first, second, third, or fourth or lower. The rank of the match was treated as categorical in analyses because there were qualitative differences between ranks that were not equivalent intervals and because the category ‘4th or lower’ captured multiple possible outcomes. Means and standard deviations are shown in Figure 1, with *F* representing the omnibus statistic associated with the ANOVA and *p* representing the statistical significance associated with the *F* statistic. These analyses revealed that the difference in happiness among groups was significant, *F*(3, 175) = 22.7, *p* < 0.001. Those who matched with their top choice program expressed the highest levels of happiness compared to those who matched with programs they ranked second, *t*(126) = 2.1, *p* = 0.04, third, *t*(109) = 6.2, *p* < 0.001, or fourth or lower, *t*(126) = 7.3, *p* < 0.001. This pattern held for excitement, *F*(3, 175) = 14.4, *p* < 0.001, and life satisfaction, *F*(3, 175) = 12.8, *p* < 0.001. Stress, a negative emotion, *F*(3, 175) = 3.8, *p* = 0.01, had the reverse pattern.

**Figure 2.**
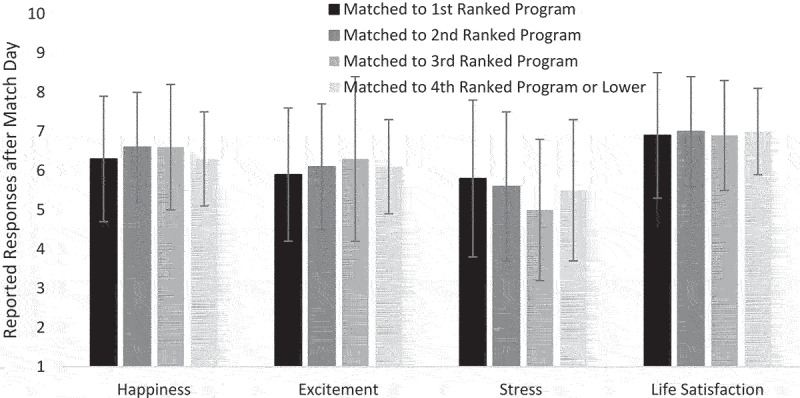
Participants rated the intensity of their responses of happiness, excitement, stress, and life satisfaction after beginning their residency program on a 9-point scale from *not at all* (1) to *most extreme possible* (9). These responses are broken out in the figure according to whether they matched with their 1st choice, their 2nd choice, their 3rd choice, or their 4th or lower choice. Values represent the mean rated intensity of each response, accompanied by the associated Standard Deviation.

At Time 3, however, several months into PGY-1, participants reported similar levels of responses regardless of the ROL position of their matched program (see Figure 2). An ANOVA revealed no significant differences among groups based on the program’s ROL position for happiness, *F*(3, 163) = 0.5, *p* = 0.7, excitement, *F*(3, 163) = 0.3, *p* = 0.9, stress, *F*(3, 163) = 0.8, *p* = 0.5, or life satisfaction, *F*(3, 162) = 0.2, *p* = 0.9.

We also analyzed the impact of perceived person-program alignment on responses shortly after Match Day (Time 2), shown in Table 3. Items shown in Table 2 were combined into program culture (Cronbach's α = 0.9), post-residency opportunities (Cronbach's α = 0.9), relationships (Chronbach’s α = 0.8), training (Chronbach’s α = 0.9), and finances (Cronbach's α = 0.7). Linear regression was used to examine the relationship between each of the factors and responses, controlling for Time 1 responses. Time 1 responses were included to account for potential individual/personality differences in the tendency to report different responses, such as happiness. Perceived alignment on program reputation predicted greater happiness, excitement, and life satisfaction, as well as greater stress. Perceived alignment on program culture predicted happiness, excitement, and life satisfaction; and negatively predicted stress, although not significantly (this relationship was significant if Time 1 stress was not a covariate). Perceived alignment on location, post-residency opportunities, relationship factors, quality of training, and finances did not predict responses.

**Table 3.** Participants rated the perceived alignment between themselves and the program on 9-point scales from *does not meet my needs at all* (1) to *perfectly suited to my needs* (9), as well as their responses shortly after Match Day on 9-point scales from *not at all* (1) to *most extreme possible* (9). The table presents the results from linear regression analyses with rated congruency on each factor predicting responses of happiness, excitement, stress, and life satisfaction shortly after Match Day. β represents the standardized beta value.

	Happiness			Excitement			Stress			Life satisfaction		
	*R*^2^ = 0.5, *F* = 21.0, *p* < 0.001			*R*^2^ =0 .5, *F* = 17.3, *p* < 0.001			*R*^2^ = 0.3, *F* = 7.0, *p* < 0.001			*R*^2^ =0.5, *F* = 18.4, *p* < 0.001		
	β	*t*	*p*	β	*t*	*p*	β	*t*	*p*	β	*t*	*p*
Reputation	0.3	3.7	0.001	0.2	2.6	0.01	0.2	1.9	.06	0.4	4.4	0.001
Location	0.1	1.1	0.3	0.1	0.9	0.4	−0.1	−1.3	.2	0.1	0.7	0.5
Program Culture	0.3	3.5	0.001	0.3	3.1	0.002	−0.2	−1.6	.1	0.2	1.9	0.05
Opportunity	−0.0	−0.2	0.8	0.0	0.0	1.0	−0.2	−1.4	.2	−0.1	−0.9	0.4
Relationship	0.2	1.9	0.1	0.1	1.5	0.1	−0.1	−0.7	.5	0.1	1.3	0.2
Training	0.0	0.3	0.8	−0.0	−0.0	1.0	−0.1	−0.6	.5	−0.0	−0.2	0.8
Finances	0.0	0.0	1.0	0.1	0.8	0.4	−0.0	−0.3	.8	0.1	0.8	0.4
Time 1	0.1	2.2	0.03	0.1	2.4	0.02	0.3	3.7	.001	0.3	5.7	0.001

We also analyzed the impact of perceived person-program alignment on responses several months into residency training (Time 3), shown in Table 4. Linear regression was again used to examine the relationship between each of the factors and responses, controlling for Time 1 responses. Perceived alignment on program reputation predicted greater life satisfaction during residency training, but not other responses. Similar to responses shortly after Match Day, perceived alignment on program culture again predicted greater happiness, excitement, and life satisfaction; and negatively predicted stress. Perceived alignment on location, post-residency opportunities, relationship factors, quality of training and finances did not predict responses.

**Table 4.** Participants rated the perceived alignment between themselves and the program on 9-point scales from *does not meet my needs at all* (1) to *perfectly suited to my needs* (9), as well as their responses after beginning their residency program on 9 point scales from *not at all* (1) to *most extreme possible* (9). The table presents the results from linear regression analyses with rated congruency on each factor predicting responses of happiness, excitement, stress, and life satisfaction after starting their residency program. β represents the standardized beta value.

	Happiness			Excitement			Stress			Life satisfaction		
	*R*^2^ = 0.2, *F* = 4.4, *p* < 0.001			*R*^2^ = 0.2, *F* = 4.5, *p* < 0.001			*R*^2^ = 0.1, *F* = 3.0, *p* < 0.004			*R*^2^ = 0.3, *F* = 9.8, *p* < 0.001		
	β	*t*	*p*	β	*t*	*p*	β	*t*	*p*	β	*t*	*p*
Reputation	0.1	0.9	0.4	0.2	1.5	0.2	0.1	0.6	0.5	0.2	2.3	0.02
Location	0.0	0.2	0.9	0.1	0.8	0.4	−0.1	−0.9	0.4	0.0	0.2	0.8
Program Culture	0.4	2.9	0.004	0.3	2.7	0.01	−0.4	−3.1	0.002	0.4	3.5	0.001
Opportunity	−0.1	−1.3	0.2	−0.2	−1.9	0.06	0.1	1.0	0.4	−0.1	−1.1	0.3
Relationship	0.2	2.0	0.8	−0.0	−0.4	0.7	0.1	1.2	0.2	0.1	0.7	0.5
Training	−0.0	−0.0	1.0	−0.0	−0.1	0.9	0.1	.6	0.5	−0.1	−.07	0.5
Finances	0.1	0.1	0.1	0.1	1.5	0.1	−0.1	−0.9	0.4	0.1	1.0	0.3
Time 1	0.1	0.8	0.4	0.1	1.8	0.1	0.2	2.7	0.01	0.3	3.6	0.001

A similar regression analysis was conducted on reported burnout at Time 3, months into their new residency program, *R*^2^ = 0.2, *F*(7, 156) = 5.0, *p* < 0.001. As with other responses, burnout was not predicted by perceived person-program alignment on the reputation of the program (β = −.2, *t* = −1.5, *p* = 0.1), location (β = −.1, *t* = −0.6, *p* = 0.6); relationship factors (β = 0.0, *t* = 0.3, *p* = 0.8), training quality (β = −.1, *t* = −0.6, *p* = 0.5), or finance factors (β = −.1, *t* = −1.6, *p* = 0.1). Perceived alignment on post-residency opportunities predicted greater burnout (β = 0.3, *t* = 2.6, *p* = 0.01), potentially due to stress related to doing well in the program when opportunities were greater. Perceived alignment on program culture predicted less burnout in the first few months of starting a residency program (β = −.3, *t* = −2.5, *p* = 0.01), accounting for approximately 30% of experienced burnout.

## Discussion

The medical residency match process is becoming more competitive, with fewer applicants matching with their top choice program every year. In 2021, only 46.4% of U.S. allopathic seniors matched with their top ranked program [20], meaning that over half of all applicants matched with a program ranked second or lower. Our findings demonstrate that matching with a top-rated program is related to being happier, more excited, and less stressed shortly after Match Day, but these emotional differences did not persist. After beginning their residency programs, residents experienced similar levels of positive and negative emotion regardless of the specific rank of their match. This finding is consistent with research showing that people often adapt quickly to positive and negative outcomes, returning to an emotional ‘baseline’ after an experience [21].

Perceived person-program alignment with the program culture of the residency program, however, did predict responses, immediately after Match Day as well as months after the start of residency. Other factors that residents reported relying on to make their rank order decisions did not predict responses after Match Day or during residency, including geographic location, training quality, relationship factors, and financial factors. After they began their residency programs, the perception that the program culture aligned with their needs predicted greater happiness and enthusiasm, and less stress and burnout. Cognitive research supports that social interactions are uniquely emotional and memorable sources of information, and have an outsize impact on decisions [21], which may make interaction-based factors like ‘rapport with program leadership’ more impactful than other factors. Taken together, these findings show that, when applicants are making ranking decisions, they undervalue factors that predict stress and burnout during residency [22].

In recent years, there has been growing concern about medical resident burnout, which affects medical trainees at higher rates than other US physicians [23,24] and is associated with negative outcomes like physician attrition [25] and increased rates of medication errors [26]. The Accreditation Council for Graduate Medical Education introduced regulations in 2003 that capped shift lengths, limited work hours, and mandated time off between shifts – at present, resident duty hours are capped at 80 hours weekly and 24 hours per continuous shift [27]. These standards aim to promote resident well-being, patient care, and education quality, though studies show that duty hour limitations alone do not improve these outcomes [28,29]. The present study demonstrates that program culture factors are also strongly linked to negative outcomes and may offer a protective or resiliency against job-related stress and burnout if there is a strong sense of person-program alignment.

Program culture factors that impact person-program alignment, such as feelings about the program, morale and rapport, life balance, caseloads and call schedules, should be considered by both medical students and residency program leaders throughout the application process. It would be beneficial for programs to discuss information related to program values and expectations with students and encourage consideration of these factors during interviews through interview questions or advising sessions prior to interviews. This has the potential to improve the quality of decisions and reduce stress and burnout among residents.

Limitations of this study include that it used a single-school sample, had a relatively small sample size, the sample of unmatched applicants was too small for analysis, some match ranks were relatively small, and it only assessed resident burnout at one timepoint. The percentage of students in our sample who matched with their top choice program (54.0%) was slightly above the national rate for U.S. allopathic seniors in 2017, 48.4%[16].

Future research should examine the link between perceived program priority alignment and emotions throughout residency[22].

## Conclusions

This study expands on existing research on decision factors in the residency match process and demonstrates that person-program alignment on program culture is important for emotional responses and burnout among residents. Although the initial emotional costs or benefits of matching with a certain program fade relatively shortly after Match Day, program culture alignment with respect to factors like call schedule and patient caseload predicted greater happiness, enthusiasm and life satisfaction, as well as lower stress and burnout in the first few months of residency. Our study provides insight into the processes involved in rank order decision-making, as well as the importance of program priorities factors for optimizing well-being and minimizing burnout during residency.
